# Enhancing bacterial survival through phenotypic heterogeneity

**DOI:** 10.1371/journal.ppat.1008439

**Published:** 2020-05-21

**Authors:** Leila M. Reyes Ruiz, Caitlin L. Williams, Rita Tamayo

**Affiliations:** Department of Microbiology and Immunology, University of North Carolina Chapel Hill, Chapel Hill, North Carolina, United States of America; Tufts Univ School of Medicine, UNITED STATES

## What are the benefits of phenotypic heterogeneity?

The ability of bacterial populations to develop phenotypically heterogeneous subpopulations has been recognized for decades. A recent study found that genetic hallmarks of phenotypic heterogeneity are ubiquitous among bacterial species of the intestinal microbiota [1], underscoring that this is a broadly employed, underappreciated survival strategy for both pathogenic and commensal bacterial species. Phenotypic heterogeneity—functional diversity among genetically identical cells—can permit division of labor and cooperative behaviors or serve as a bet-hedging strategy to help ensure the survival of the population as a whole. Division of labor occurs when biological processes are separated among subpopulations, conferring an overall fitness advantage to the population. For example, the opportunistic pathogen *Pseudomonas aeruginosa* forms multiple unique cell types that perform specialized roles in the formation of biofilm. Some cells contribute to biofilm extracellular matrix production and initiate biofilm development, while others exhibit surface motility and allow biofilm expansion [2]. In the bet-hedging strategy, incorporating phenotypic variants into a population increases the likelihood that some members will survive a stress. In settings where environmental changes are rapid and stark, phenotypic heterogeneity helps ensure the survival of the population at the expense of individuals not well suited to the current environment, which are selected against and lost. This strategy is advantageous over traditional sense-and-respond mechanisms, which require an adaptation time that may be insufficient in the face of sudden or severe environmental changes. Mechanisms of phenotypic heterogeneity may be particularly important in the context of disease, as these mechanisms are more common in host-associated bacteria than in aquatic or terrestrial species [1].

## How does phase variation result in phenotypic heterogeneity?

Phase variation is among the best characterized processes of generating phenotypic heterogeneity. Phase variation typically involves an ON and OFF switch in a phenotype that contributes to fitness in one environment but that may have a fitness cost in a different condition [3]. A number of genetic mechanisms that result in phase variation of a gene have been described, including slipped-strand mispairing in repetitive nucleotide tracts, DNA inversions by site-specific recombinases, and allele shuffling by recombination (reviewed in [4]). These mechanisms have in common stochasticity, heritability, and reversibility, allowing the bacterial population to exhibit and regenerate phenotypic heterogeneity.

The insertion or deletion of nucleotides by slipped-strand mispairing during replication of repetitive DNA tracts, known as short sequence repeats (SSR), is one means of altering gene expression. Variations in the number of nucleotides within an SSR in a coding sequence may cause a frameshift mutation that creates a premature stop codon and a truncated product (OFF) or, conversely, may restore the reading frame and production of the gene product (ON). Changes in an SSR within a promoter can alter transcription factor binding sites, leading to changes in the transcription initiation of a gene.

**Fig 1:**
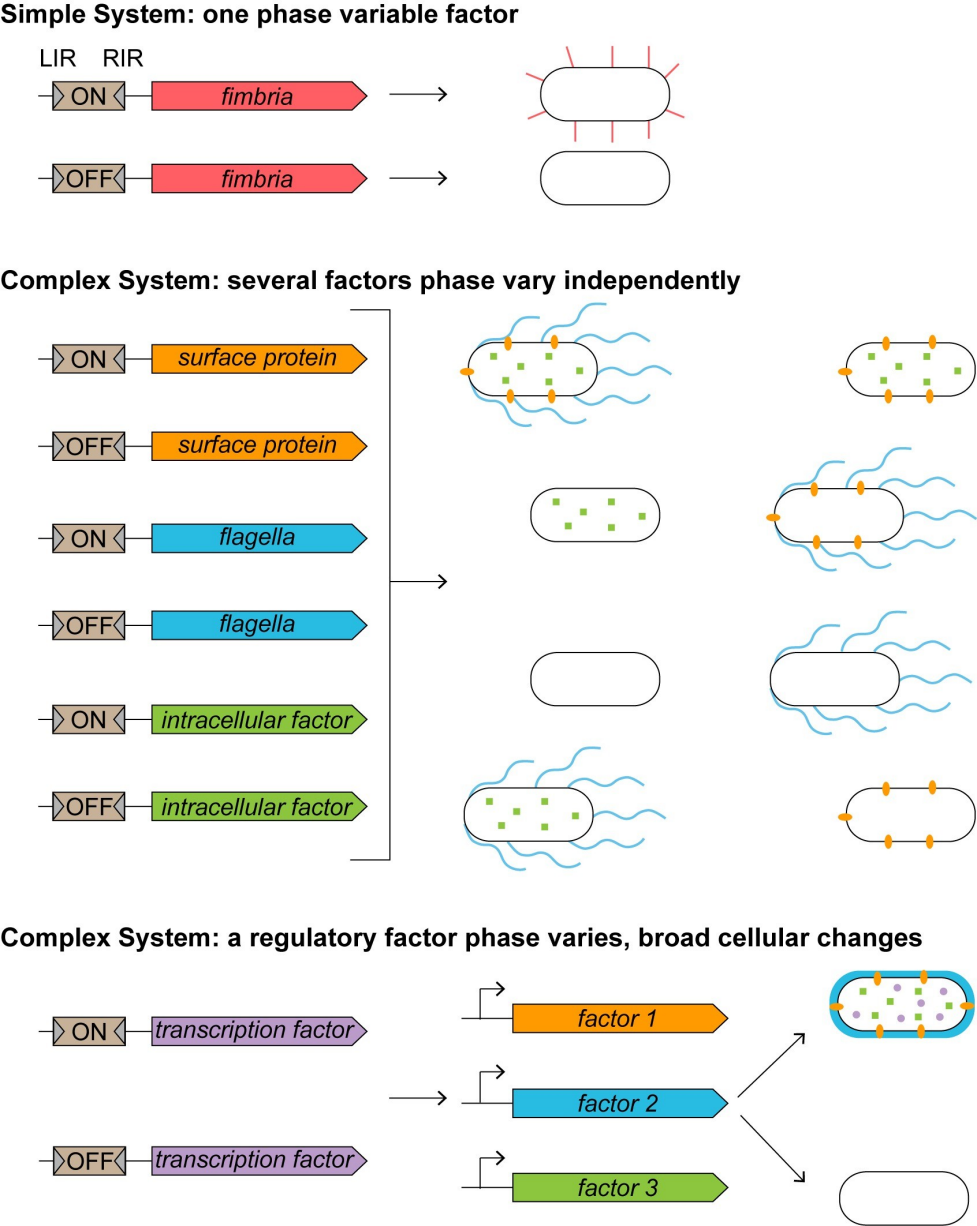
Phase variation can result in singular versus global regulation of cellular factors. Phase variation via site-specific DNA recombination involves the inversion of a regulatory sequence by a serine or tyrosine recombinase. The recombinase binds flanking inverted repeats (LIRs and RIRs) and catalyzes the inversion of the intervening sequence. The orientation of the invertible element determines whether the downstream gene(s) are expressed (ON) or not (OFF). Top: In a simple system, recombination inverts a single element, controlling the expression of a single factor. Fimbrial phase variation in *Escherichia coli* is controlled in this manner. Middle: In a more complex scenario, a single recombinase inverts multiple elements. Inversion of the elements occurs stochastically and independently, yielding multiple discrete genetic combinations and phenotypic variants, thus allowing one recombinase to influence bacterial phenotypes on a broader scale. As an example, in *C*. *difficile*, the recombinase RecV inverts sequences upstream of multiple genes including those encoding CwpV, flagella, and intracellular factors [19, 22, 31]. Bottom: Phase variation of a single regulator, such as a transcription factor, results in a coordinated switch in multiple phenotypes regulated by that factor. LIR, left inverted repeat; RIR, right inverted repeat.

Phase variation by site-specific DNA recombination is mediated by a site-specific recombinase that binds to inverted repeats and catalyzes inversion of the sequence between the repeats. The invertible sequence itself contains regulatory information that, when properly oriented, impacts the expression of an adjacent gene or operon (Fig 1, top). The best characterized mechanisms involve an invertible element containing a transcriptional promoter that initiates expression when oriented toward a coding sequence (ON) but not when inverted (OFF) [5, 6]. Alternatively, the orientation of the invertible element may regulate a factor by a post-transcriptional mechanism, as has been suggested for phase variation of the cell wall protein CwpV and flagella in *Clostridioides difficile* [7, 8]. Finally, phase variation can be achieved by shuffling alleles of the same gene between an expressed locus and a distal, silent locus. This mechanism results in a heterogeneous yet clonal bacterial population expressing different allelic variants of a factor.

Regardless of mechanism, the phenotypic makeup of the population is driven by environmental selection, often leading to a strong bias in the population for a specific variant. After cessation of a selective pressure, phenotypic heterogeneity can be restored, typically within several generations. Though phase variation events are stochastic, regulation of factors that influence the rate of switching impacts phase variation and the population makeup [9].

## What are common regulatory targets of phase variation?

Phase variation typically regulates the production of complex structures, such as flagella, pili, fimbriae, and exopolysaccharides [3]. These structures are exposed on the bacterial surface and directly interface with the cell’s environment. In the case of bacterial pathogens, these factors are thought to promote infection by contributing to colonization (e.g., pili) or evading immune clearance (e.g., capsule). However, production of these factors comes at a cost, including the energy cost of biosynthesizing complex macromolecules and/or the cost of host innate immune recognition (e.g., flagella). Given the cost and benefit trade-offs, the dynamics of phase variation are attuned to the environmental niches and pressures experienced by individual bacteria through the course of an infection.

Phase-locked mutants that cannot phase vary, e.g. due to mutation of a recombinase gene or inverted repeats, are valuable tools for defining the role of phase variation of a factor. In *E*. *coli*, phase variation of type 1 fimbriae occurs through site-specific recombination of a promoter-containing invertible element upstream of the fimbrial subunit gene, *fimA* [10]. These fimbriae promote colonization of the bladder epithelium in uropathogenic *E*. *coli* [11]. Consistent with this result, phase-locked OFF mutants lacking fimbriae are attenuated in disease models of urinary tract infection [12, 13]. A more complicated regulatory scheme involving an invertible promoter regulates the transcription of alternate flagellin genes in *Salmonella enterica*. In both *E*. *coli* and *S*. *enterica*, while the surface structure positively impacts aspects of pathogenesis, the specific role of phase variation is unclear—why employ phase variation rather than typical regulatory mechanisms? Presumably, as described above, heterogeneity provides protection against rapid environmental changes and immune recognition. Investigating the spatial and temporal dynamics of phase variation of these structures during the course of infection could reveal additional information about the roles of these factors as well as allow inferences about the host selective pressures impacting the population.

## How does phase variation mediate global changes in gene expression?

In addition to ON and OFF switching of the production of surface factors, phase variation can broadly affect bacterial physiology. Some DNA methyltransferases, enzymes that methylate specific nucleotide motifs, are subject to phase variation, resulting in a population of bacteria with different methylation patterns. Because methylation can influence gene expression, methyltransferase phase variation can influence the transcription of multiple genes simultaneously. Such phase variable regulons, often referred to as phasevarions, include genes involved in colonization and pathogenesis in several pathogens [14, 15]. *Streptococcus pneumoniae* contains six alleles encoding the type I methyltransferase SpnD39III HsdS (SPND39IIIA-F). Recombination-mediated shuffling between the *hsdS* alleles results in expression of different allelic variants and heterogeneity in genomic methylation patterns within the population, impacting the expression of virulence genes, such as capsular biosynthesis genes [16].

Phase variation of transcriptional regulators similarly can lead to global changes in gene transcription (Fig 1, bottom). For example, *Bordetella pertussis* switches between virulent and avirulent states through the modulation of the BvgAS two-component system (TCS) [17]. The *bvgS* gene contains a cytosine tract in its coding region and SSR-mediated variation in the number of cytosines affects BvgS production and transcription of BvgAS-regulated virulence genes [17, 18]. In *C*. *difficile*, two invertible elements lie upstream of the colony morphology regulator genes *cmrRST* and the flagellar *flgB* operon, which each encode transcriptional regulators [8, 19]. The *cmrRST* operon encodes a noncanonical TCS composed of a predicted histidine kinase, CmrS, and two putative response regulators with DNA binding domains, CmrR and CmrT [19]. CmrRST modulates multiple *C*. *difficile* phenotypes: colony morphology, biofilm formation, swimming motility, cell shape, and virulence in a model of acute disease [19]. Phase variation of CmrRST therefore has the capacity to coordinately regulate genes involved in these phenotypes. The 23 kb *flgB* operon contains the alternative sigma factor gene *sigD*, which is therefore also subject to phase variation [8]. In addition to coordinating the transcription of flagellar genes, SigD promotes the transcription of the genes encoding the toxins, TcdA and TcdB, and a number of other genes [20, 21]. Thus, phase variation of SigD concomitantly controls production of flagella, toxins, and more.

Five additional invertible elements that flip by site-specific recombination have been identified in *C*. *difficile* R20291 [7, 8, 19, 22]. Two are present upstream of genes encoding phosphodiesterases (PDEs) that degrade the cyclic diguanylate (c-di-GMP) signaling molecule, which is known to control the transition between free-living and adherent, sessile states in numerous bacterial species, including *C*. *difficile* [23, 24]. Hypothetically, phase variation of these PDEs could cause heterogeneity in intracellular c-di-GMP levels among individual bacteria and, consequently, in c-di-GMP regulated phenotypes such as swimming motility [25].

Interestingly, several of the targets of phase variation in *C*. *difficile* share a common feature: Inversion of the upstream DNA element is mediated by a single tyrosine site-specific recombinase, RecV [22]. Inversion of multiple, distinct sequences by RecV provides *C*. *difficile* with a mechanism to introduce extensive heterogeneity into the population (simplified in Fig 1, middle). Such a large pool of variants may improve the survival of *C*. *difficile* in the intestinal tract. Similarly, in the intestinal opportunistic pathogen *Bacteroides fragilis*, the site-specific recombinase Mpi mediates inversion of seven invertible elements containing promoters that regulate genes involved in the production of different capsular exopolysaccharides [26].

## How does the host environment influence phenotypic heterogeneity?

Many important human pathogens phase vary factors that influence their virulence [27], and new studies highlight the impact of the phenomenon on important nosocomial pathogens. Recent work by Tipton and colleagues describes the phase variation of *Acinetobacter baumannii* between two distinct colony morphologies. These variants, termed opaque and translucent, differ in a number of in vitro phenotypes, including motility, biofilm formation, cell shape, antibiotic resistance, capsule production, and resistance to immune components [28, 29]. Additionally, the opaque variant is more virulent than the translucent in two infection models, wax moth larvae (*Galleria mellonella*) and murine pneumonia [28, 29]. *A*. *baumannii* isolates obtained from blood cultures of five patients yielded only opaque colonies, suggesting that the opaque variant is selected during human bloodstream infection [29]. The underlying phase variation mechanism remains to be characterized but correlates with expression of an OmpR/EnvZ TCS orthologue and the expression of a TetR family transcriptional regulator [29, 30]. Because *A*. *baumannii* exists in both the environment and host, the opaque phenotype may be beneficial in the context of infection, but not in the external environment. Alternatively, the translucent variant may be beneficial in other host contexts. *A*. *baumannii* can colonize a number of body sites, and the phenotypically distinct variants may be better suited to a particular niche. This concept of higher fitness of certain phase variants in particular infection sites has been demonstrated for *Haemophilus influenzae*, where certain phase variable genes are OFF in one infection site (e.g., middle ear infection) but ON in another context, e.g., invasive disease [3].

As noted above, phase variation of the CmrRST in *C*. *difficile* impacts multiple phenotypes, including virulence [19]. Phase switching between variants was shown to occur in vivo; a CmrRST phase-ON inoculum converted to a predominantly phase-OFF population by the endpoint of infection [19]. These results imply that the phase-ON state is detrimental to *C*. *difficile* in this infection model. However, the high conservation of the *cmrRST* locus across *C*. *difficile* strains suggests that this regulatory system confers some benefit. An analysis of the spatial and temporal distribution of *cmrRST*-expressing cells during infection could shed light on the shifts in CmrRST heterogeneity during infection and on the host selective pressures impacting the population.

Determining the molecular basis and fitness benefits of phase variation and phenotypic heterogeneity in bacterial pathogens will provide insight into pathogenic mechanisms and may influence the development of preventive and therapeutic options. For example, if a vaccine or therapeutic target is phase variable, then the pathogen may escape the immune response or treatment. However, if we understand the selective pressures leading to population shifts or block the variation mechanism, we may be able to ensure the entire population remains vulnerable, improving drug efficacy and patient outcomes.
